# Immobilised teicoplanin does not demonstrate antimicrobial activity against *Staphylococcus aureus*

**DOI:** 10.1038/s41598-022-20310-8

**Published:** 2022-10-05

**Authors:** S. Britton, K. Lee, L. Azizova, G. Shaw, W. Nishio Ayre, J. P. Mansell

**Affiliations:** 1 Department of Applied Sciences, University of the West of England, Coldharbour Lane, Bristol, BS16 1QY UK; 2 Department of Chemistry, Green-Nano Materials Research Center, Kyungpook National University, Daegu, 41566 South Korea; 3 School of Dentistry, Cardiff University, Cardiff, CF14 4XY UK

**Keywords:** Biochemistry, Microbiology, Medical research, Materials science

## Abstract

Antibacterial bone biomaterial coatings appeal to orthopaedics, dentistry and veterinary medicine. Achieving the successful, stable conjugation of suitable compounds to biomaterial surfaces is a major challenge. A pragmatic starting point is to make use of existing, approved antibiotics which are known to remain functional in a stationary, immobilised state. This includes the macrocyclic glycopeptide, teicoplanin, following the discovery, in the 1990’s, that it could be used as a chiral selector in chromatographic enantiomeric separations. Importantly teicoplanin works at the level of the bacterial cell wall making it a potential candidate for biomaterial functionalisations. We initially sought to functionalise titanium (Ti) with polydopamine and use this platform to capture teicoplanin, however we were unable to avoid the natural affinity of the antibiotic to the oxide surface of the metal. Whilst the interaction between teicoplanin and Ti was robust, we found that phosphate resulted in antibiotic loss. Before contemplating the covalent attachment of teicoplanin to Ti we examined whether a commercial teicoplanin stationary phase could kill staphylococci. Whilst this commercially available material could bind N-Acetyl-L-Lys-D-Ala-D-Ala it was unable to kill bacteria. We therefore strongly discourage attempts at covalently immobilising teicoplanin and/or other glycopeptide antibiotics in the pursuit of novel antibacterial bone biomaterials.

## Introduction

Minimising infection risk of implantable bone biomaterials, e.g., titanium (Ti), continues to be a priority area in contemporary materials science research^1^. Covalent grafting of suitable antibacterial agents to Ti could be a potential route but finding solutions to creating antibacterial surfaces is especially challenging; selected agents need to retain functionality when in a stationary, immobile state and need to demonstrate biocidal activity by acting at the level of the cell wall or membrane^2^. Evidence of stability to γ-irradiation typically applied to medical devices is also highly desirable. Importantly the antimicrobial agent should display low or minimal toxicity, demonstrating compatibility with host cells to ensure osseointegration. In realising the development of an antibacterial Ti technology, we have taken inspiration from enantioselective chromatography using covalently immobilised glycopeptide antibiotics as the chiral selectors^3–9^.

**Figure 1:**
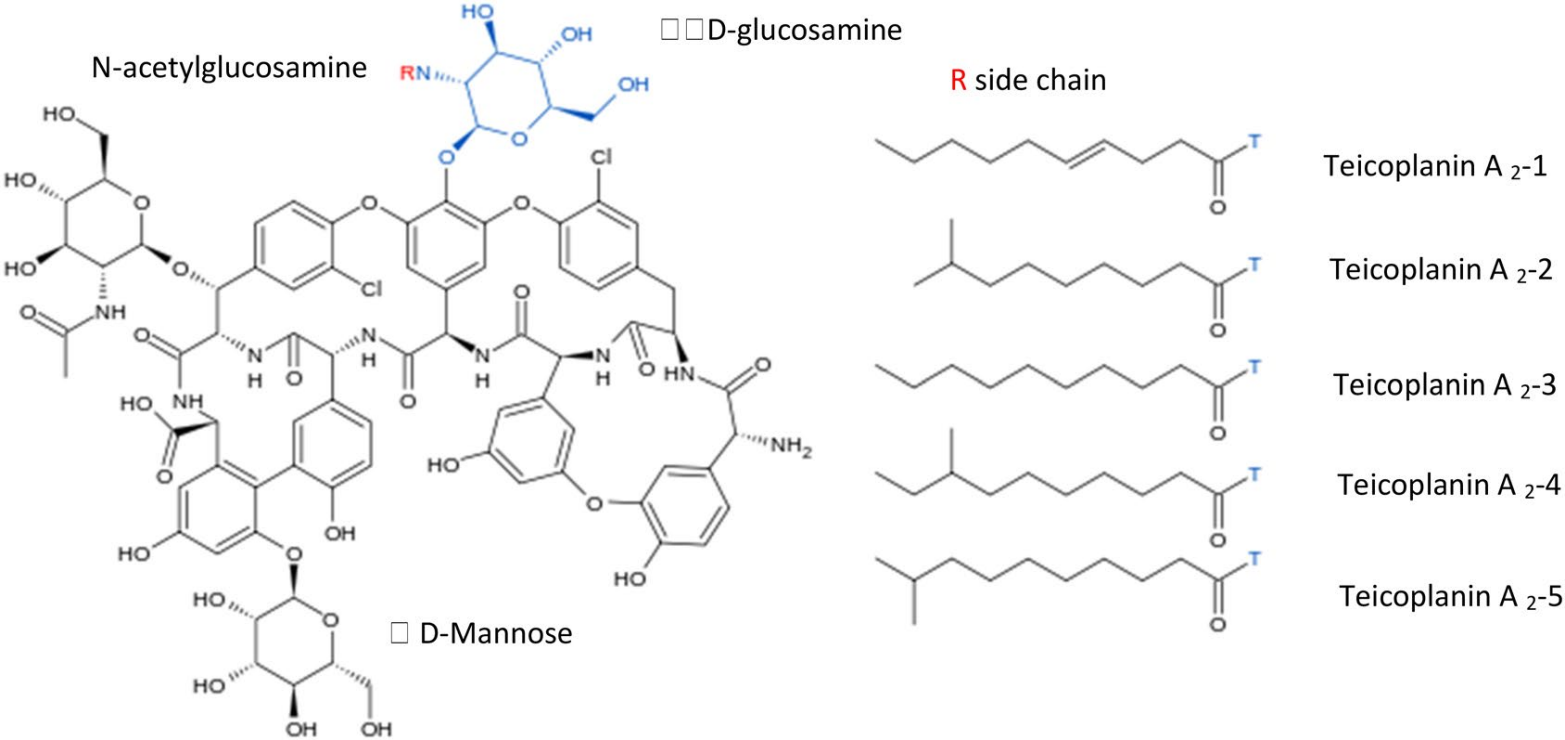
Structure of teicoplanin.

With regard to sourcing the appropriate antibacterial agent we have focused on teicoplanin (Teic), a macrocyclic glycopeptide antibiotic (Fig. 1) produced by *Actinoplanes teichomyceticus*^10^. Teic binds to the C-terminal of D-Ala-D-Ala motifs of non-crosslinked lipid II which forms during the biosynthesis of peptidoglycan, an essential component of the bacterial cell wall. This is especially important in realising the fabrication of a stably bound antibacterial Ti coating because Teic is acting at the level of the cell wall to eradicate bacteria. Both anaerobic and aerobic Gram-positive bacteria are susceptible to Teic, including MRSA which has particular significance in the context of total joint replacement infections^1^. Compared to other glycopeptide antibiotics, e.g., ristocetin and vancomycin, Teic is markedly more surface active with multiple, unique, functional groups. Teic can be likened to a semi-rigid aglycone basket composed of four main ring systems that are fused. These in turn consist of seven aromatic rings, two of which have chlorine-substitutions and four of which exhibit ionisable phenolic moieties. Within the aglycone basket is an amine (cationic site) juxtaposed to a carbonyl group and a carboxylic acid group (anionic site). At the periphery are three monosaccharides; α-D-mannose, β-D-N-acetylglucosamine and β-D-N-acylglucosamine of which one of the glucosamine residues is tethered to a fatty N-acyl chain extension. It is the presence of the hydrocarbon chain that is peculiar to Teic and a feature enabling the antibiotic to nestle within the lipid leaflet of bacteria^11^.

During the early to mid-1990’s Teic became a powerful tool in the development of novel chiral stationary phases for chromatographic enantiomeric separations^12^. As a covalently-bound chiral selector, Teic performs well in the reversed phase, normal phase and "polar-organic” modes yielding highly efficacious multimodal chromatography media^12^. In more recent times Teic bonded to silica is considered a “state of art” material for utilisation in super/subcritical liquid chromatography^13^. Specialised chromatography columns packed with silica covalently bonded to Teic for enantiomeric separations have been commercially available for many years, for example TeicoShell from Azyp LLC (Arlington, Texas).

The discovery that covalently tethered, immobilised Teic could retain biological function is key in developing antibacterial bone biomaterials that are capable of directly killing MRSA. Compared to vancomycin, Teic has a greater stability and exhibits lower nephro/ototoxicity and has reduced potential for causing red-man syndrome^14^. With regard to obvious concerns over antibiotic resistance there have been some noteworthy developments in Teic biochemistry. For example, Pathak and Miller^15^ have chemically modified the antibiotic to develop brominated variants to help combat the risk of resistance by providing a greater spectrum of antibacterial products. There is also evidence that Teic can withstand 25 kGy of γ-irradiation^16^, the preferred sterilisation route for implantable devices. Collectively these features place Teic as a choice candidate for Ti-functionalisation.

We initially sought to develop an antibacterial Ti surface by first coating the metal with polydopamine (PDA) following a report on the successful generation of a magnetic chiral stationary phase using Teic conjugated to PDA-coated iron oxide particles^17^. During the course of our work, we were unable to successfully graft Teic to PDA-Ti and found that the antibiotic simply bound well to the natural oxide finish of the metal. Some of the findings from this work are presented herein. We next turned our attention to covalently binding Teic to Ti following earlier claims that this might be a viable option for glycopeptide antibiotics^18,19^. Before doing so we wanted to ascertain if a commercially available chiral stationary phase, consisting of Teic covalently tethered to silica shells, was capable of killing staphylococci.

## Materials and methods

### Reagents and Ti disc preparation

Unless stated otherwise all reagents were of analytical grade and purchased from Sigma-Aldrich (Poole, UK). Orthopaedic-grade Ti discs (10 mm diameter; 1.5 mm thickness) were kindly provided by OsteoCare (Slough, UK). On arrival the discs were thoroughly cleaned by extensive distilled water washing, exposure to acetone, water rinsing again, followed by concentrated nitric acid exposure, a further extensive water wash and then baking, in air, at 180 °C for 72 h. 14 mm TiO_2_-coated AT cut QCM sensors (Ti-QCM, Ti/Au metallization with a TiO_2_ coating and resonant frequency of 5 MHz) were purchased from Microvacuum (Budapest, Hungary).

### Bacterial culture preparation

The organism used was a *Staphylococcus aureus* strain (NCTC 12981) obtained from Public Health England (Salisbury, UK). Stock cultures were maintained on beads at − 80 °C. Working cultures were maintained on Brain–Heart Infusion agar plates (BHIA; Oxoid, Basingstoke, UK) and sub-cultured weekly for a maximum of four weeks to maintain viability and colony characteristics. For broth inoculum preparation, 2–3 colonies were taken from a BHIA plate into 10 ml Brain–Heart Infusion broth (BHIB; Oxoid, Basingstoke, UK) and incubated for 16–18 h at 37 °C with gentle shaking (120 RPM). The reason for using BHIB is that it is a good, rich, general-purpose medium that permits good growth of a variety of bacteria. Standards were made by adjusting overnight cultures in phosphate buffered saline (PBS) to an OD_625nm_ of 0.08–0.12 (0.5 McFarland standard; approx. 1.5 × 10^7^ CFU/ml) and further diluted in a 1% Petone/PBS broth to a final density of 1.5 × 10^5^ CFU/ml. This was used in all microbiological assays unless specified. Since Teic is active on the cell wall of proliferating cells, the bacteria need to be metabolically active, hence the rationale for using a minimal medium supplemented with 1% peptone.

### Bacterial viability to Teic-Ti

This method was adapted from Ayre et al.^20^. Ti discs were immersed in 500 µg/ml of Teic (1 ml) in 2-(N-Morpholino)ethanesulfonic acid (MES; 50 mM; pH 5.47) for 2 h, washed in distilled water twice to remove weakly bound Teic and allowed to dry. Once dried, Ti discs were exposed to a 10^5^ CFU/ml suspension of *S. aureus* for 24 h at 37 °C with gentle shaking (120 RPM). After incubation, discs were recovered, washed twice with 0.85% saline and sonicated for 5 min in 1 ml of maximum recovery diluent (MRD; Oxoid, Basingstoke, UK) to recover attached bacteria. MRD is an isotonic diluent that combines a protective effect of peptone with the osmotic balance of physiological saline. The detached bacteria were transferred to a sterile universal tube and incubated for 30 min at 37 °C in order to achieve maximum recovery. The rationale for taking this approach is to reduce the risk of bacterial loss from sonication when using purely aqueous media. The recovered organisms were diluted and dispensed onto BHIA using the Miles and Misra technique^21^ and incubated for 24 h at 37 °C prior to colony counting. Over a period of approximately 2 months a total of 12 independent experiments were performed using 36 control and 36-functionalised Ti discs.

### X-ray photoelectron spectroscopy (XPS) of Teic-Ti

In parallel with the studies conducted above, Ti discs were exposed to 500 µg/ml solution of Teic in 50 mM MES, pH 5.47 and left, under ambient conditions from 30 min up to 3 h. To analyse elements on the surface of Ti and functionalised samples, XPS spectra were taken by using NEXSA XPS system (ThermoFisher, Waltham, MA, US). A monochromatic X-ray source (Al-Kα) beam was used for the data collection. The calculation of the atomic percentages was performed using Avantage Data System software (ThermoFisher, Waltham, MA, US).

### Teic-Ti adsorption studies

Orthopaedic-grade Ti discs and solid TiO_2_ powder (mixture of rutile and anatase) were used to examine Teic adsorption to the metal oxide.

For the TiO_2_ powder, 50 mg was dispensed into microcentrifuge tubes and immersed in 500 µl of Teic (500 µg/ml in 50 mM HEPES; pH 7.4) and left to incubate, for up to 30 min, under ambient conditions. TiO_2_ with HEPES only was used as a control. At each time point, samples were centrifuged at 11,300 × g for 2 min and 25 µl of each supernatant was added to 500 µl of freshly prepared bicinchoninic acid reagent (BCA; ThermoFisher, Waltham, MA, US) as per the manufacturer’s instructions and incubated for 30 min at 60 °C. After incubation, sample aliquots (100 µl) were subsequently transferred to a 96-well plate and measured against a series of Teic standards at 540 nm using a microplate reader (TECAN, Männedorf, Switzerland). To confirm Teic-TiO_2_ attachment, the supernatant was decanted and the powder samples were washed twice by centrifugation and resuspended in 500 µl HEPES buffer. A 25 µl sample of each powder suspension was added to 500 µl of BCA reagent, incubated and assessed in the same manner as above.

### Assessment of binding robustness between Teic and TiO_2_

To determine how robust the Teic adsorption was to the TiO_2_, TiO_2_ powder (50 mg) was added to microcentrifuge tubes, followed by 500 µl of Teic (500 µg/ml) in HEPES (50 mM; pH 7.4) buffer and incubated under ambient conditions for 2 h. TiO_2_ with HEPES buffer only was used as a control. Once incubated, samples were centrifuged at 11,300 × g for 2 min, the supernatants were discarded*,* and the pellets were washed up to 10 times with HEPES buffer (500 µl per wash). After 1, 3, 5 and 10 washes, 25 µl of each wash was combined with 500 µl of BCA and incubated for 30 min at 60 °C. Once incubated, 100 µl aliquots were transferred to a 96-well plate and measured at 540 nm using a microplate reader (TECAN, Männedorf, Switzerland). To assess the efficacy of the Teic-TiO_2_ attachment, the supernatant was decanted after each wash and remaining pellets resuspended in 500 µl of HEPES buffer. A 25 µl sample of the resuspended pellet was combined to 500 µl of BCA reagent and incubated for 30 min at 60 °C. Once incubated, samples were centrifuged to pellet the powder and the supernatant was measured in the same manner as above.

Thermal Gravimetric Analysis (TGA) was performed in order to assess the thermal stability of immobilised Teic to the oxide of TiO_2_. TiO_2_ powder (50 mg) was added to microcentrifuge tubes, followed by addition of either 500 µl of Teic (500 µg/ml) in HEPES (50 mM; pH 7.4) buffer or HEPES buffer alone as a control. The samples were incubated under ambient conditions for 2 h and then centrifuged at 11,300 × g for 2 min. The supernatant was discarded and the pellets air dried at room temperature. Teic powder was also analysed as a reference sample. Samples were run on a Pyris 1 TGA under a nitrogen flow of 40 ml/min (Perkin Elmer, Beaconsfield, UK), The sample was stabilised at 30 °C for 20 min before heating to 800 °C at a rate of 5 °C/min. Data was analysed using Pyris TGA software (Perkin Elmer, Beaconsfield, UK).

### Influence of phosphate on Teic-TiO_2_ binding

The impact of phosphate on the bonding between Teic-TiO_2_ was also examined given the affinity of the anion to Ti^22^. Briefly TiO_2_ powder (50 mg) were exposed to 500 µg/ml Teic prepared in PBS (pH 7.3), where the phosphate concentration was either 1 mM or 10 mM and samples left to incubate at room temperature for 30 min. Teic (500 µg/ml)-TiO_2_ in HEPES (50 mM; pH 7.4) and TiO_2_ in HEPES only were used as controls. After incubation, samples were centrifuged and 25 µl of the supernatant was combined to 500 µl BCA reagent and incubated for 30 min at 60 °C. Once incubated, 100 µl aliquots were transferred to a 96-well plate and measured at 540 nm using a microplate reader. To confirm Teic-TiO_2_ attachment, the supernatant was decanted, the powder samples were washed once by centrifugation (11,300 × g) for 2 min and TiO_2_ pellets resuspended in 500 µl buffer. A 25 µl aliquot of each powder suspension was added to 500 µl of BCA reagent and incubated for 30 min at 60 °C. Once incubated, powder samples were centrifuged and 100 µl of each supernatant were transferred to a 96-well plate and measured using a microplate reader.

### Influence of phosphate on Teic detachment from TiO_2_

To investigate the impact of phosphate on the detachment of Teic from TiO_2_, a quartz crystal microbalance (QCM) system with impedance measurement was used (QCM-I, MicroVacuum, Budapest, Hungary). TiO_2_-coated QCM sensors (Ti-QCM) were subjected to HEPES buffer (50 mM; pH 7.4) at a flow rate of 100 µl/min using a peristaltic pump (Ismatec Reglo Digital, Wertheim, Germany) at a constant temperature of 21 °C using a high precision built in Peltier driver to avoid temperature drifts (stability of ± 0.02 °C). After a stable baseline was obtained over a minimum of 10 min, 500 µg/ml Teic in HEPES was injected using a Rheodyne MXP injection system with a semiautomatic switching valve (MXP9960-000, California, US) for another 10 min. The Ti-QCM sensor was then subjected to a final 10 min with either HEPES or PBS with 1 mM, 2 mM, 5 mM or 10 mM phosphate concentrations. Frequency at the 1st, 3rd and 5th overtones and dissipation measurements were collected. Sauerbrey mass was calculated using Biosense software (Microvacuum, Budapest, Hungary). Experiments were performed in triplicate.

### Teic-Ti elution studies

Ti discs were exposed to Teic (500 µg/ml in 50 mM MES, pH 5.4) and incubated for 2 h under ambient conditions. After incubation, discs were washed with distilled water to dislodge any loosely bound Teic and allowed to dry in a sterile environment. Control and functionalised Ti discs were then immersed in 1% Peptone/PBS and left for 24 h at 37 °C with gentle shaking (120 RPM). After incubation, the recovered conditioned media (100 µl) was transferred to a sterile 96-well plate and inoculated with *S. aureus* (100 µl) to achieve a final density of 10^5^ CFU/ml. The samples were then incubated at 37 °C for a further 24 h. At the desired time the optical density OD at 595 nm (using a microplate reader) was taken to ascertain bacterial growth in the conditioned media. To determine if the conditioned discs still retained any antimicrobial activity the control and functionalised Ti discs were transferred to a new multiwell plate and washed in distilled water twice, allowed to dry and then exposed to a 10^5^ CFU/mL suspension of *S. aureus* and incubated for a further 24 h at 37 °C with gentle shaking (120 RPM). After incubation, discs were recovered, washed twice with 0.85% saline and sonicated for 5 min in 1 ml of MRD to recover the attached bacteria. The detached bacteria were transferred to a new universal and incubated for 30 min at 37 °C in order to achieve maximum recovery. The recovered organisms were diluted and dispensed onto BHIA using the Miles and Misra technique^21^ and incubated for 24 h at 37 °C prior to colony counting.

### Teico stationary phase (TSP)—N-Acetyl-L-Lys-D-Ala-D-Ala binding studies

A commercially available chiral stationary phase, consisting of covalently tethered Teic to silica shells (TSP) was sourced from AZYP (Arlington, Texas) in which approximately 68 mg of Teic is bound to 1 g of silica. The tripeptide N-Acetyl-L-Lys-D-Ala-D-Ala (Cambridge Bioscience, UK) was reconstituted to 200 µg/ml in 50 mM HEPES (pH 7.4). Briefly, TSP (100 mg) was exposed to 500 µl of the tripeptide.

(200 µg/ml) and incubated under ambient conditions for 30 min. After incubation, the sample was centrifuged (11,300 × g) for 2 min and the supernatant was collected (500 µl) and transferred into a new microcentrifuge tube. The pH of the supernatant was adjusted to ~ 8.4 using NaOH and exposed to 5 µl of genipin (250 mM in DMSO) and then left to react at 60 °C for 2 h. To assess the binding of the tripeptide to the TSP, the remaining pellet was washed three times in HEPES (50 mM; pH 7.4) using centrifugation and resuspended in 500 µl of HEPES buffer. The pH of the pellet was adjusted to ~ 8.4 and subsequently exposed to 5 µl of genipin and left to react for 2 h at 60 °C. After incubation, the OD_595_ of the samples were measured using a microplate reader (TECAN, Männedorf Switzerland). Plain silica shells (AZYP, TX, US) was used as a control and assessed in the same manner.

### Bacterial viability to TSP

To assess the antibacterial activity of the chiral stationary phase, aliquots (50 mg) were added to microcentrifuge tubes and immersed in a 70% ethanol solution for 10 min to sterilise the powder. This was washed three times in a 1% peptone/PBS solution using centrifugation (11,300 × g). After centrifugation, the washed pellets were resuspended in 1 ml of a 1% peptone/PBS solution containing 10^5^ CFU/ml of *S. aureus* and transferred to a sterile bijou. A further 4 ml of the bacterial suspension was added to the bijou to a final volume of 5 ml (final concentration of TSP suspension was 10 mg/ml, corresponding to a final Teic concentration of 680 µg/ml). This was incubated for 24 h at 37 °C with gentle shaking (120 RPM). A final concentration of free teicoplanin (600 µg/ml) and untreated silica were used as controls. After incubation, samples were diluted in MRD, dispensed onto BHIA using the Miles & Misra technique^21^ and incubated for a further 24 h at 37 °C prior to colony counting.

### Statistical analysis

Unless stated otherwise, all experiments above were repeated at least three times in triplicate. All data were subject to a normality test to ensure data were normally distributed. A one- or two-way analysis of variance (ANOVA) or Kruskal–Wallis test was used, where appropriate, to test for statistical significance using Graphpad Prism software (Version 8; San Diego, CA, USA) with *p* < 0.05 regarded as being statistically significant. When *p* ≤ 0.05 was found, a Tukey or Sidak’s multiple comparisons post-test was used between all groups. In some instances, an unpaired *t*-test (2-tailed) were used to compare the means of functionalised and control surfaces. All data are expressed as the mean together with the standard deviation.

## Results

### Ti discs exposed to Teic exhibits antibacterial activity

**Figure 2:**
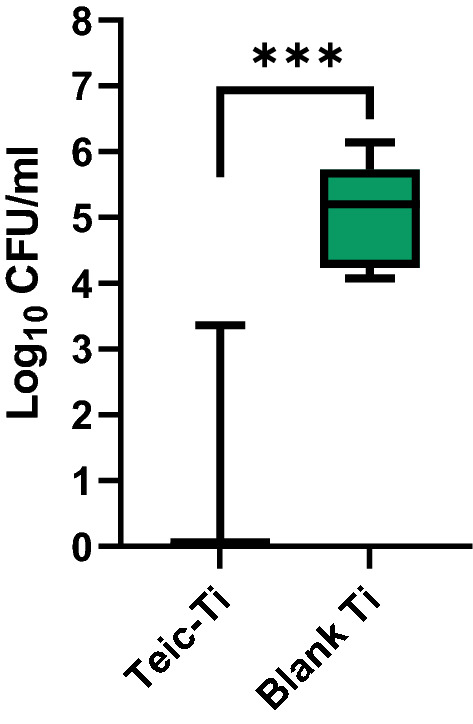
Antibacterial efficacy of Teic-modified Ti. Ti discs were immersed in 500 µg/ml Teic (50 mM MES; pH 5.47) for 2 h under ambient conditions. After incubation, functionalised and control discs were transferred to a new well plate, washed twice in distilled water and subsequently exposed to a 10^5^ CFU/ml suspension of *S. aureus* in 1% Peptone/PBS for 24 h at 37 °C with gentle shaking (120 RPM). After incubation, discs were recovered, washed twice with 0.85% saline and sonicated for 5 min in 1 ml of MRD to recover attached bacteria. The detached bacteria were transferred to a new universal and incubated for 30 min at 37 °C in order to achieve maximum recovery. The recovered organisms were diluted and dispensed onto BHIA and incubated for 24 h at 37 °C. When Ti discs are steeped in a solution of Teic the modified surfaces are capable of significantly reducing bacterial attachment in comparison to the control (blank) Ti (***p < 0.0001). All data (mean ± SD; N = 36) represent pooled triplicate runs from 12 independent experiments.

Bacterial attachment to Teic-modified Ti was assessed by exposing *S. aureus* to functionalised and control Ti discs for 24 h at 37 °C. Attachment of *S. aureus* to the modified Ti surface was found to be significantly lower (*p* < 0.0001) when compared to Ti alone (Fig. 2) and resulted in a 5-log reduction from the initial seeding density (10^5^ CFU/ml). Of the 36 Ti discs exposed to Teic, only 7 specimens were associated with modest bacterial growth (~ 10^2^–10^3^ CFU/ml). Similar results were obtained for Ti discs exposed to Teic for as little as 5 min (data not shown).

### Physiochemical evidence of Teic adsorption to Ti

**Figure 3:**
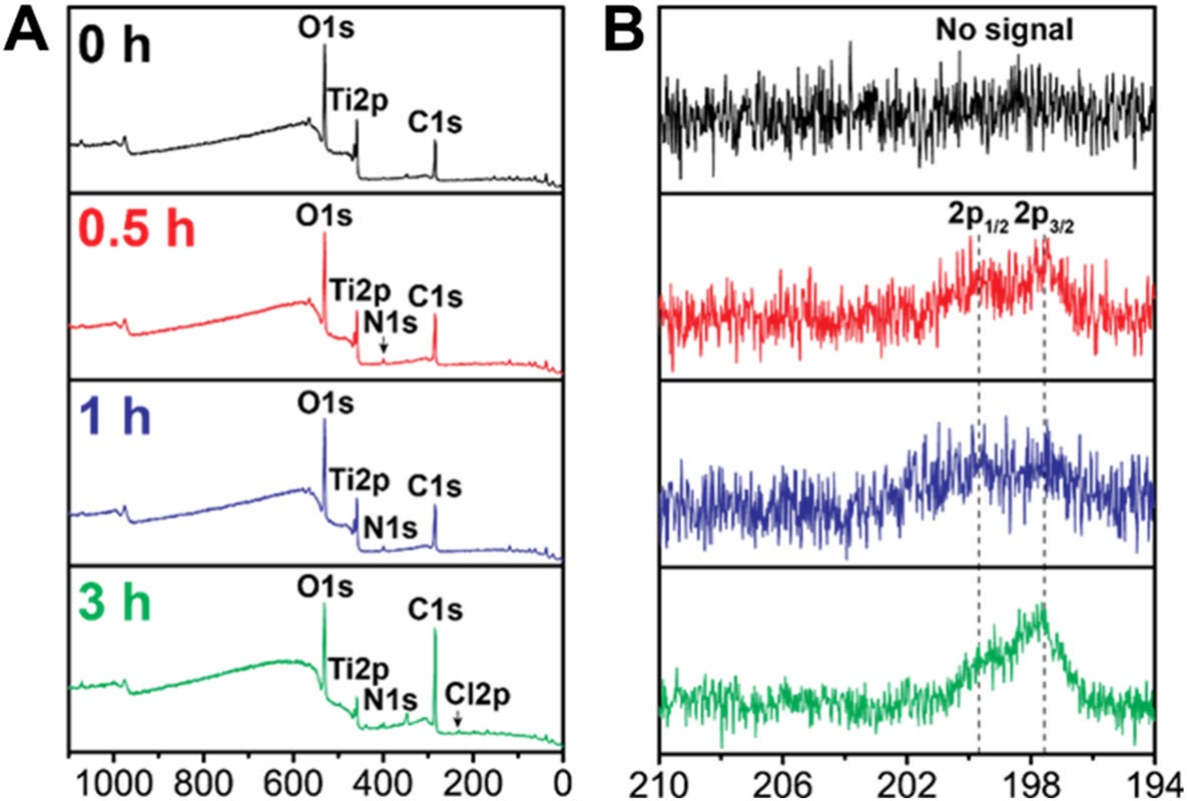
Detection of Teic coating on the Ti surface. X-Ray Photoelectron Spectroscopy (XPS) analysis of (**A**) survey peaks and (**B**). high-resolution Cl2p peaks of Teic-treated Ti discs with different immersion times: 0, 0.5, 1, and 3 h. It is clear that Teic adsorption occurs within 30 min of Teic exposure.

To analyse elements on the surface of control and functionalised Ti samples, XPS was performed. Confirmation of the presence of Teic was observed in the form of increasing chlorine peaks (Cl_2_) over time, suggesting that Teic can adsorb to the surface within 30 min of exposure to the antibiotic (Fig. 3). As expected, a change in the atomic composition with increasing time supported adsorption of the antibiotic to Ti (Table 1).

**Table 1:** Elemental composition of control and Teic-functionalised Ti.

Time (h)	C1s (at.%)	N1s (at.%)	O1s (at.%)	Cl2p (at.%)	Ti2p (at.%)
–	41.9 ± 1.9	1.4 ± 0.1	45.0 ± 1.6	–	11.7 ± 0.4
0.5	45.0 ± 1.0	2.5 ± 0.2	43.2 ± 0.8	0.3 ± 0.1	9.1 ± 0.5
1	47 ± 0.6	2.6 ± 0.5	41.4 ± 0.9	0.3 ± 0.1	8.8 ± 0.1
3	62.3 ± 0.4	3.0 ± 0.1	30.1 ± 0.5	0.5 ± 0.1	4.0 ± 0.5

Quantified atomic percentage (at.%) of the elements in each Ti specimen treated with Teic. The clear changes in elemental composition with increasing incubation time support Teic adsorption to the metal surface.

### Teic binds avidly to TiO_2_

**Figure 4:**
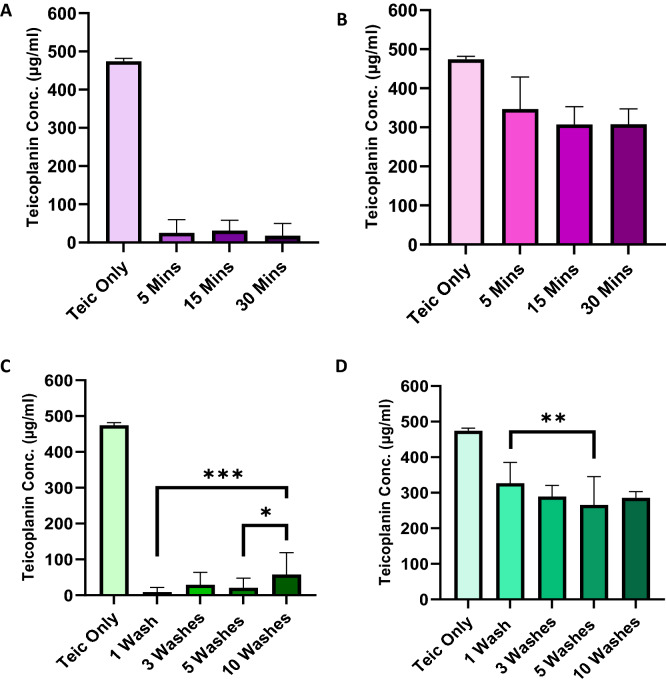
Evidence of a rapid and robust adsorption of Teic to TiO_2_. (**A**) TiO_2_ powder (50 mg) was treated with 500 µl of 500 µg/ml Teic in 50 mM HEPES (pH 7.4) and incubated at room temperature for 5, 15 and 30 min. At each time point, samples were centrifuged at 11,300 × g for 2 min and 25 µlof each supernatant was combined to 500 µl of BCA reagent. Samples were incubated for 30 min at 60 °C and the absorbances read at 540 nm against an incubation control (Teic only). Within 5 min there is evidence of antibiotic adsorption to TiO_2_. (**B**) To confirm Teic-TiO_2_ adsorption, the supernatant was decanted, and samples were washed twice by centrifugation and resuspended in 500 µl HEPES buffer. A 25 µl aliquot of each powder sample was added to 500 µl of BCA reagent and incubated for 30 min at 60 °C. Once incubated, powder samples were centrifuged at 11,300 × g for 2 min and 100 µl of each sample were transferred to a 96-well plate and measured in the same manner as above. (**C**) TiO_2_ powder (50 mg) were similarly treated with 500 µl of Teic and incubated at room temperature for 2 h. Once incubated samples were centrifuged at 11,300 × g for 2 min, the supernatants were discarded, and the pellets were washed up to 10 times with HEPES buffer (500 µl). Aliquots (25 µl) of each wash were subject to the BCA assay to ascertain antibiotic leaching. (**D**)After each set of washes the remaining TiO_2_ pellets were resuspended in fresh buffer (500 µl) and samples containing the powder (25 µl) taken for the BCA assay. All data (mean ± SD; N = 20) represent pooled quadruplicate runs from 5 independent experiments.

To observe the adsorption of Teic to the metal oxide, TiO_2_ powder was used. Interestingly, it was found that Teic adsorption to TiO_2_ was the same for all time points tested (Fig. 4A,B) and would suggest that immersing the oxide in Teic for as little as 5 min affords functionalisation. To determine the durability of Teic attachment to TiO_2_ the modified oxide powder was subjected to repeated washings. Despite the small loss of Teic after the first wash (Fig. 4C,D) the oxide still retained a large amount of the antibiotic, even after 10 washes, suggesting a robust attachment.

**Figure 5:**
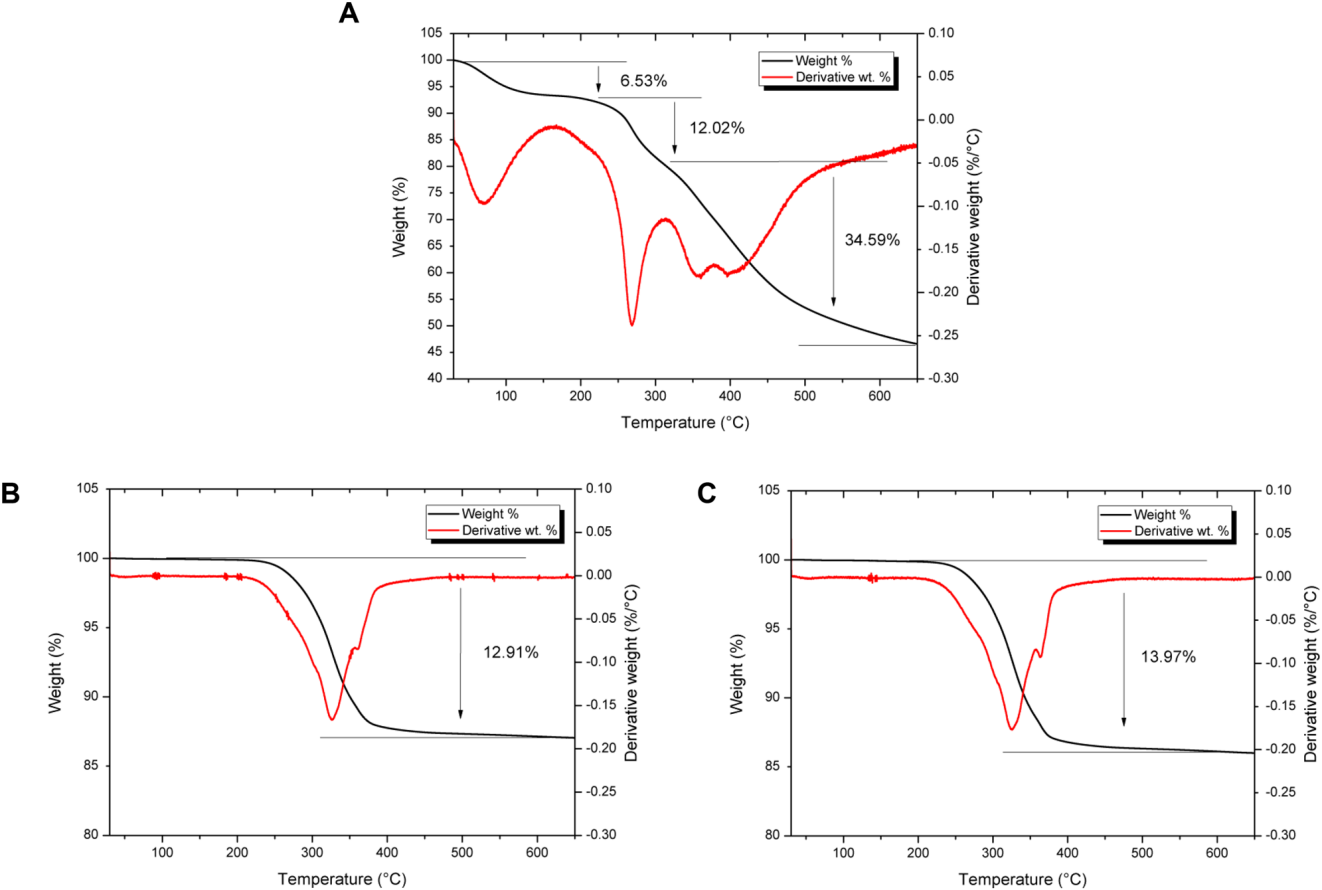
Thermal stability of Teic immobilised on the surface of TiO_2_ using Thermal Gravimetric Analysis (TGA). TiO_2_ powder (50 mg) was incubated with either 500 µl of Teic (500 µg/ml) in HEPES (50 mM; pH 7.4) buffer or HEPES buffer alone as a control for 2 h under ambient conditions and then centrifuged at 11,300 × g for 2 min. The centrifuged pellets were air dried at room temperature before undergoing TGA. (**A**) Percentage weight curves for Teic alone shows an initial decrease at temperatures below 100 °C due to moisture evaporation. This was followed by rapid thermal degradation at temperatures greater than 250 °C. (**B**) TiO_2_ samples incubated in HEPES buffer showed a single dissociation step between 250 °C and 400 °C and a percentage weight change of 12.91%. (**C**)Similar percentage weight and derivative weight curves were obtained for TiO_2_ samples incubated with Teic, however a greater weight loss of 13.97% was observed between 250 °C and 400 °C indicating the additional dissociation of Teic from the surface of TiO_2_ at high temperatures.

To further elucidate the robustness of the Teic attachment to the oxide of TiO_2_, TGA was performed on Teic alone, TiO_2_ powder and TiO_2_ powder functionalised with Teic. Percentage weight loss curves for Teic alone showed an initial decrease at temperatures below 100 °C due to residual moisture evaporation caused by the hydrophilic nature of Teic. Thermal decomposition of Teic was observed at temperatures greater than 250 °C with three distinct derivative peaks demonstrating maximum weight loss rates at approximately 270 °C, 357 °C and 402 °C (Fig. 5A). TiO_2_ powder incubated in HEPES buffer showed a single weight loss step between 250 °C and 400 °C and a total percentage weight reduction of 12.91% (Fig. 5B). Similar percentage weight and derivative weight curves were obtained for TiO_2_ samples incubated with Teic, however a greater weight loss of 13.97% was observed between 250 and 400 °C with a more distinct derivative peak at 365 °C (Fig. 5C). This greater weight loss indicates the dissociation of Teic from the surface of TiO_2_ at high temperatures and thermal stability of immobilised Teic at temperatures up to 250 °C.

### Phosphate compromises the binding of Teic to TiO_2_

**Figure 6:**
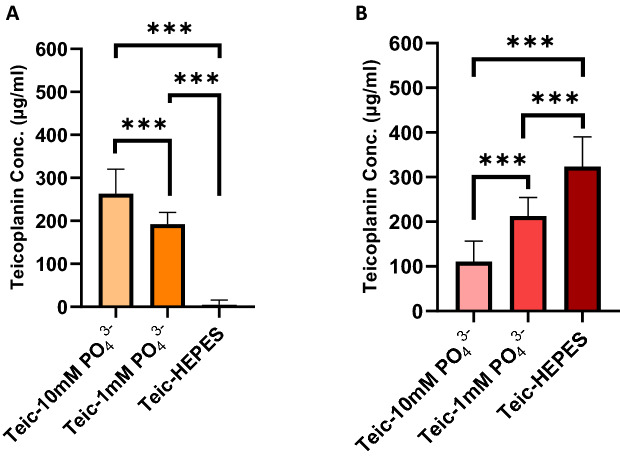
Influence of phosphate on Teic adsorption to TiO_2_. (**A**) TiO_2_ powder (50 mg) were exposed to 500 µg/ml Teic in PBS (500 µl) where the phosphate concentration was either 1 mM or 10 mM and samples left to incubate at room temperature for 30 min. Teic (500 µg/ml) reconstituted in 50 mM HEPES (pH 7.4) served as a positive control for TiO_2_ binding. After incubation, 25 µl aliquots of the supernatant were combined to 500 µl BCA reagent and incubated for 30 min at 60 °C. Once incubated, 100 µ aliquots were transferred to a 96-well plate and measured at 540 nm using a microplate reader. It is evident that phosphate reduces the extent of Teic binding to the metal oxide (***p < 0.0001). (**B**) To confirm Teic attachment to TiO_2_, the supernatant was decanted, and the powder samples were washed once by centrifugation (11,300 × g) and resuspended in 500 µl buffer. A 25 µl sample of each powder suspension was added to 500 µl of BCA reagent and incubated for 30 min at 60 °C. Once incubated, samples were centrifuged and 100 µl of the supernatant were transferred to a 96-well plate and measured in the same manner as above. Significantly less Teic (***p < 0.0001) is present on TiO_2_ exposed to phosphate. All data (mean ± SD; N = 20) represent pooled quadruplicate runs from 5 independent experiments.

The impact of phosphate on the bonding between Teic and TiO_2_ was examined as the anion is known to have a strong affinity for the oxide^22^. It was found that phosphate could compromise the binding of Teic to TiO_2_ and that increasing the phosphate concentration from 1 to 10 mM resulted in less antibiotic binding to the oxide (Fig. 6). This likely suggests that the phosphate anion has a greater affinity for the oxide than the antibiotic. To control for the possible influence of sodium and/or chloride ions we reconstituted Teic in 50 mM HEPES (pH 7.4) supplemented with NaCl up to 1 M and found this to be without impact on antibiotic binding to TiO_2_ (data not shown).

### Phosphate displaces Teic bound to TiO_2_ rapidly

**Figure 7:**
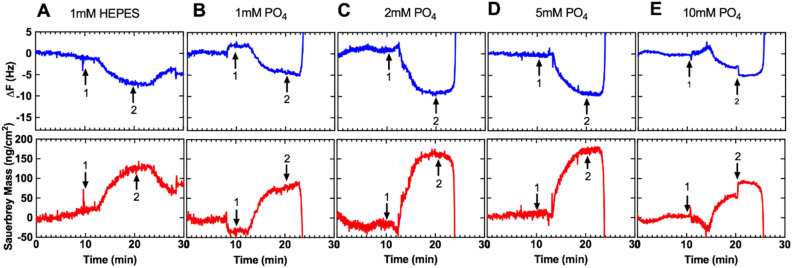
Influence of phosphate on Teic detachment from TiO_2_*.* Ti-QCM sensors were subjected to HEPES buffer (50 mM; pH 7.4) at a flow rate of 100 µl/min at 21 °C for 10 min prior to 500 µg/ml Teic in HEPES for 10 min (arrow 1). Coated Ti-QCM sensors were then washed for a final 10 min with either (**A**). HEPES or PBS with (**B**). 1 mM, (**C**). 2 mM, (**D**). 5 mM or (**E**). 10 mM phosphate concentrations (arrow 2). Representative plots from triplicate (N = 3) experiments showing change in fundamental frequency (upper panels) and calculated Sauerbrey mass (lower panels).

The ability of phosphate to readily detach Teic from the surface of TiO_2_ is shown in Fig. 7. Injection of 500 µg/ml Teic in HEPES resulted in rapid adsorption of Teic to the surface of the Ti-QCM sensor which plateaued after approximately 5 min, resulting in a surface coverage of 100–200 ng/cm^2^ (based on Sauerbrey mass calculations). Washing the surface of the coated Ti-QCM sensors with HEPES resulted in removal of loosely bound Teic (Fig. 7A) however the majority of Teic still remained on the surface of the Ti-QCM sensor. Conversely, washing the surface with phosphate containing buffers resulted in the immediate dissociation of Teic from the Ti-QCM sensor (Fig. 7B–E). Slight delays between injections and changes in resonant frequency were observed due to the length of tubing and time required for the injected sample to reach the surface of the Ti-QCM sensor.

### Evidence of Teic loss following culture conditioning of Teic-functionalised Ti

**Figure 8:**
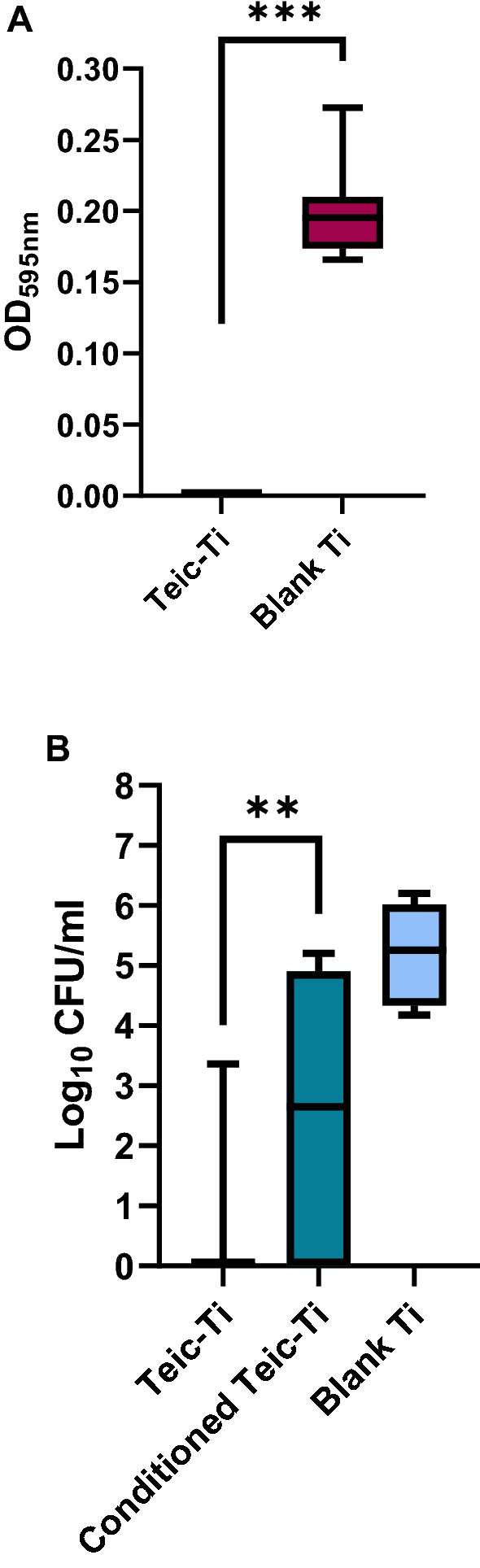
Antibacterial activity of Teic-Ti after culture conditioning. (**A**). Ti discs were exposed to Teic (500 µg/ml in 50 mM MES, pH 5.4) and incubated for 2 h under ambient conditions. After incubation, discs were washed with distilled water to dislodge any loosely bound Teic and allowed to dry in a sterile environment. Control and functionalised Ti discs were then immersed in 1% Peptone/PBS and left for 24 h at 37 °C with gentle shaking (120 RPM). After incubation, the recovered conditioned media (100 µl) was transferred to a sterile 96-well plate and inoculated with *S. aureus* (100 µl) and left to incubate at 37 °C for 24 h. At the desired time the OD at 595 nm was measured to ascertain bacterial growth using a microplate reader. The significant reduction (*** *p* < 0.0001) in OD supports antibiotic leaching into the culture medium (**B**). To determine if the conditioned discs still retained any antimicrobial activity the control and functionalised Ti discs were transferred to a new multiwell plate and washed in distilled water twice, allowed to dry and then exposed to a 10^5^ CFU/mL suspension of *S. aureus* and incubated for a further 24 h at 37 °C with gentle shaking (120 RPM). After incubation, discs were recovered, washed twice with 0.85% saline, and sonicated for 5 min in 1 ml of MRD to recover the attached bacteria. The detached bacteria were transferred to a new universal and incubated for 30 min at 37 °C in order to achieve maximum recovery. All data (mean ± SD; N = 9) represent pooled triplicate runs from 3 independent experiments.

To determine if the conditions of bacterial culture result in antibiotic elution from the Ti surface, functionalised and control Ti discs were subject to a mock bacterial culture for 24 h. As anticipated the culture media recovered from control Ti supported bacterial growth with evidence of solution turbidity and OD_595_ values ranging between 0.17 and 0.27 (Fig. 8A). In contrast, the conditioned media recovered from Teic-functionalised Ti completely inhibited bacterial growth, as indicated by clear solutions and OD_595_ values similar to the blanks. To assess the bactericidal activity, media recovered from Teic-Ti was spot inoculated on to BHIA and left to incubate for 24 h at 37 °C. This confirmed that the culture medium from the modified discs exhibited a large amount of bactericidal activity as no growth occurred after incubation when compared to the culture medium recovered from the control Ti discs (data not shown). The conditioned discs were subsequently exposed to *S. aureus* to determine if they were still able to kill bacteria. The findings obtained for these conditioned samples indicated a loss of antibacterial activity, further confirming that the antibiotic elutes from the metal surface under the conditions described (Fig. 8B).

### A Teic chiral stationary phase (TSP) binds to the tripeptide N-Acetyl-L-Lys-D-Ala-D-Ala

**Figure 9:**
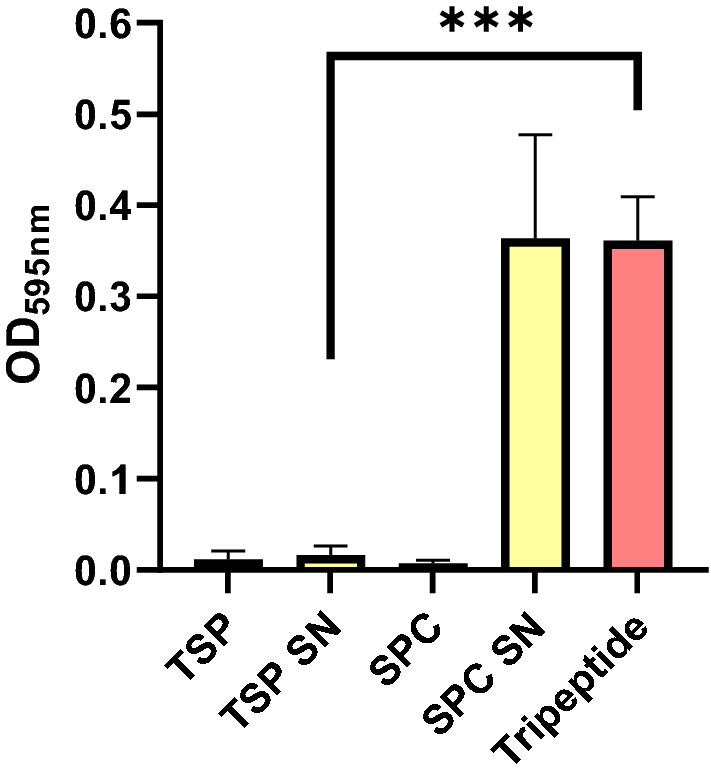
The tripeptide N-Acetyl-L-Lys-D-Ala-D-Ala binds avidly to a teicoplanin stationary phase (TSP). Aliquots (100 mg) of TSP and a silica particle control (SPC) were exposed to 500 µl of the tripeptide (200 µg/ml) prepared in 50 mM HEPES (pH 7.4) and incubated at room temperature for 30 min. After incubation, samples were centrifuged and the supernatants (SN) collected, the pH adjusted to 8.4 and 500 µl treated with 5 µl of genipin (250 mM in DMSO). Samples were left to incubate at 60 °C for 2 h. After incubation, the optical density (OD) of the samples were measured using a microplate reader. The OD for the tripeptide control and SPC supernatants were similar indicating no tripeptide binding to the SPC. In contrast the supernatant recovered from the TSP had a significantly reduced OD (***p  < 0.0001) when compared to the tripeptide control, indicating good binding of the tripeptide with the TSP. All data (mean ± SD; N = 9) represent pooled triplicate runs from 3 independent experiments.

The binding of the chiral stationary phase to the tripeptide was monitored using genipin, a natural chemical component that forms a blue chromogen when it reacts with the ε-amino group of lysine^23^. The results indicate that the tripeptide successfully bound to TSP (Fig. 9). The OD_595_ of the tripeptide control and the silica particle control (SPC) supernatants were essentially similar, suggesting that none of the tripeptide bonded to the SPC. In contrast, the TSP supernatant had a significant decrease in OD_595_ (Fig. 9), suggesting the tripeptide was able to bind well to the TSP. As expected, these findings provide evidence of a functional chiral stationary phase.

### Immobilised Teic does not demonstrate biocidal activity against *S. aureus*

**Figure 10:**
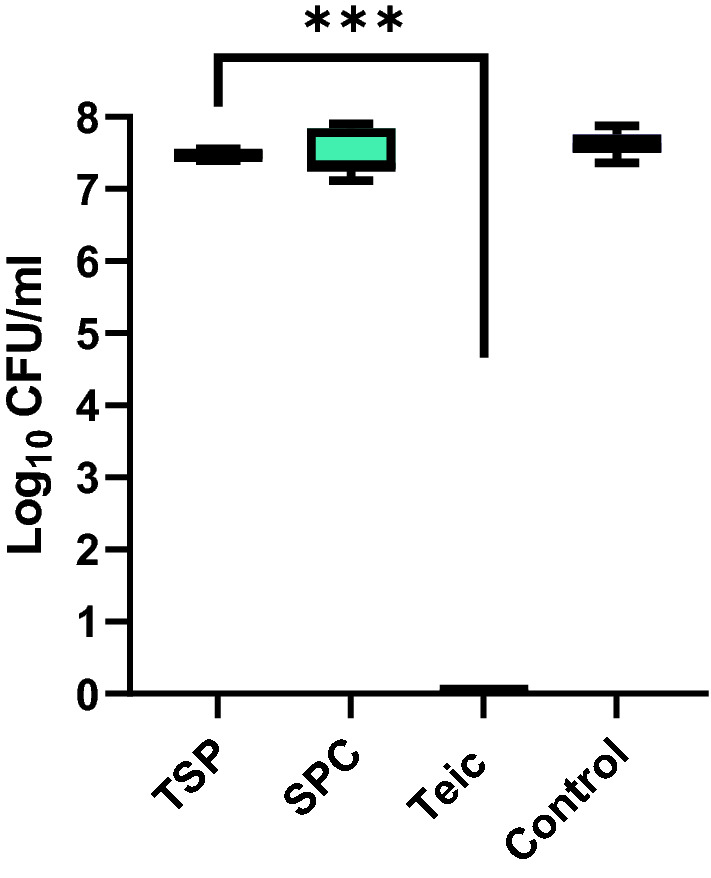
Immobilised Teic does not demonstrate biocidal activity against *S. aureus*. Aliquots (50 mg) of TSP and a silica particle control (SPC; 50 mg) were exposed to a 10^5^ CFU/mL suspension of *S. aureus* and incubated for 24 h at 37 °C. A bacterial culture alone and a culture containing free Teic (600 µg/ml) served as controls. As anticipated, cultures treated with free Teic led to complete bacterial demise (***p < 0.0001). In contrast, the TSP cultures exhibited similar bacterial numbers to SPC and bacterial controls. All data (mean ± SD; N = 9) represent pooled triplicate runs from 3 independent experiments.

To determine if immobilised Teic, as a chiral stationary phase, had the capacity to kill bacteria, both the TSP and SPC were exposed to *S. aureus* for 24 h. When *S. aureus* (10^5^ CFU/ml) was exposed to free Teic (600 µg/ml), there was the expected, stark bactericidal activity when compared to the bacterial control which resulted in a ≥ 7-log reduction from the initial inoculum density (Fig. 10). However, when *S. aureus* was exposed to 10 mg of TSP (with a Teic concentration equivalent to 680 µg/ml), there was no effect on bacterial viability when compared to the SPC and inoculum control. These findings indicate that when Teic is covalently tethered to a surface it is incapable of killing bacteria.

## Discussion

The persistence of total joint replacement infections has a significant socioeconomic impact on both patient and healthcare providers and has incentivised the scientific community and industry to develop novel technologies to combat this problem. The covalent grafting of suitable antimicrobials could be a potential solution to minimising infection risk. In doing so the selected agent is firmly fixed to the implant surface thereby securing increased residence time and reducing the risk of antimicrobial leaching into the surrounding environment. Immobilised antimicrobials must therefore act at the level of the cell wall and/or membrane^2^ and display evidence of functionality when tethered to a surface. One such candidate is Teic, a macrocyclic glycopeptide antibiotic, which has found widespread use as a chiral selector in enantiomeric chromatography^3–9,12,13^ and attacks Gram-positive bacteria at the level of the cell wall^10^.

During the course of our studies, we found that simply steeping medical-grade Ti discs in an aqueous solution of Teic generated a modified surface, as supported by XPS analysis and accompanying changes in the atomic composition of the metal surface. Importantly these Teic-functionalised discs demonstrated antimicrobial activity against *S. aureus*. This is in keeping with a report by Aykut and colleagues^16^ who treated Ti wires with a methanolic solution of Teic, the resultant material of which was capable of killing *S. aureus* and preventing Ti infection in a rabbit model. Titania (TiO_2_) is the natural surface finish of the metal and a compound widely used as a chromatography sorbent for the enrichment and purification of glycopeptides^24–26^.

To corroborate that Teic could bind to TiO_2_ we exposed a mixture of rutile and anatase oxide (50 mg) to an aqueous solution of Teic (500 µl; 500 µg/ml) and monitored antibiotic binding using a BCA reagent. Adsorption of the glycopeptide antibiotic to TiO_2_ was rapid; within 5 min approximately 90% was captured by the oxide. This was further demonstrated through QCM measurements. Furthermore, the interaction was robust, with the majority of the antibiotic remaining bound after multiple (up to 10) washings or continuous flow in HEPES buffer in the case of the QCM data. The attachment was also thermally stable as shown by the TGA data where dissociation of Teic from the TiO_2_ surface was only observed at temperatures greater than 250 °C. We also found that Teic remained bound to TiO_2_ even when left incubated under neutral conditions for a week at 37 °C (data not shown). However, when Teic-functionalised Ti discs were subjected to a mock bacterial culture the conditioned media exhibited antibacterial activity. It was clear that the antibiotic was leaching from the metal surface, and this was the reason why the functionalised Ti was able to kill *S. aureus*. We subsequently found that phosphate could displace Teic from Ti discs and that the anion could compromise the binding of Teic to TiO_2_. This is most likely attributed to the fact that phosphate binds strongly to TiO_2_^22^.

Whilst the facile adsorption of Teic to Ti might be a convenient solution towards the development of an antibacterial finish it was evident that this interaction was compromised by physiological phosphate (~ 1 mM) and therefore not an option towards tackling the issue of total joint replacement infections. Our findings echo those of Aykut and colleagues^16^ wherein Teic leaching was evident from treated Ti wires, as indicated from their agar diffusion assays. An alternative strategy was clearly needed, and we considered covalent grafting of the antibiotic to Ti given that immobilised Teic is able to function as a chiral selector in enantiomeric chromatography settings.

Herein we confirm that a commercially available teicoplanin chiral stationary phase (TSP), as expected, bound well to N-Acetly-L-Lys-D-Ala-D-Ala, a peptide that shares similarity to the D-Ala-D-Ala motifs of non-crosslinked staphylococcal undecaprenyldiphospho-N-acetylmuramoyl-[N-acetylglucosamine]- L-alanyl-γ-D-glutamyl-L-lysyl-D-alanyl-D-alanine^27^. The selection of N-Acetyl-L-Lys-D-Ala-D-Ala to confirm TSP functionality was informed from a study by Economou et al*.*^27^ who successfully used this tripeptide for the detection of Teic via a direct fluorescence polarisation assay. We were able to monitor tripeptide binding to the TSP using genipin which reacted with the ε-amino group of the lysine residue producing a dark blue chromogen^23^. The binding between the TSP and the tripeptide was compelling with sample supernatants having undetectable levels of the tripeptide within 30 min. This was in stark contrast to control silica particles exposed to the tripeptide wherein recovered supernatants reacted with genipin producing deep blue solutions. Whilst we were able to confirm TSP functionality, interestingly this same material did not demonstrate antimicrobial activity against *S. aureus*. Indeed, the equivalent of approximately 680 µg/ml Teic, as bound to silica, was without effect on bacterial viability. In our hands we typically find that the treatment of these bacteria with free Teic at a final concentration of 50 µg/ml results in the expected, complete bacterial demise within 24 h (data not shown). It is unlikely that the Teic of the TSP is unable to target bacterial D-Ala-D-Ala because spacers or linkers are used to couple the antibiotic to the silica shells; in the case of the TSP used in this study, the silica is initially functionalised with (3-aminopropyl)triethoxysilane (APTES) followed by 1,6 diisocyanatohexane to enable Teic-functionalisation of the pre-treated silica^28^. Thus the antibiotic is away from the surface of this commercially available TSP.

Our findings conflict with other studies reporting on the successful, covalent, functionalisation of Ti and iron oxide nanoparticles (IONP’s) with Teic/glycopeptide antibiotics^18,19^. In each instance APTES was used to coat the surface with amines followed by NHS-EDC chemistry to conjugate the carboxyl of Teic to the amine at the metal surface. However, these reports did not provide any physiochemical evidence, e.g., using XPS to confirm that Teic was tethered to the metal surface through the proposed mechanism of binding. In addition, no studies were forthcoming to ascertain the robustness of the interaction between the surface and the antibiotic by performing mock bacterial cultures or leaching experiments. These considerations are particularly important given that glycopeptides bind avidly to TiO_2_^25,26,29^.

It is quite possible that the aforementioned studies^18,19^ are inadvertently reporting on the natural and strong affinity of Teic/glycopeptide antibiotics to the metal oxides and that these functional materials, as in our hands, are capable of demonstrating activity against *S. aureus* because of antibiotic leaching into the culture media. It would seem most likely that the phosphate present in the culture media used in these studies promotes antibiotic leaching from the metal surface. In contrast, a commercial, covalently functionalised Teic-chiral stationary phase, which interacted well with N-Acetly-L-Lys-D-Ala-D-Ala, exhibited no antibacterial activity. To conclude we strongly advise against finding ways of covalently grafting glycopeptide antibiotics to Ti and, indeed, any (bio)material surface in the pursuit of novel antibacterial devices.

## Data Availability

The datasets used and/or analysed during the current study are available from the corresponding author (Jason.mansell@uwe.ac.uk) on reasonable request.
